# Association between triglyceride glucose index and glaucoma: results from the NHANES 2005–2008

**DOI:** 10.1097/MD.0000000000044934

**Published:** 2025-10-03

**Authors:** Zongxiu Luo, Jianye Chen, Lijuan Wei

**Affiliations:** a College of Traditional Chinese Medicine, Changchun University of Chinese Medicine, Changchun, Jilin Province, China; b The Affiliated Hospital of Changchun University of Chinese Medicine, Changchun, China.

**Keywords:** chronic inflammation, glaucoma, insulin resistance, NHANES, nonlinear relationship, triglyceride-glucose index

## Abstract

Glaucoma is a leading cause of irreversible vision loss and represents a substantial global public health challenge. Growing evidence implicates metabolic dysregulation (particularly insulin resistance) in the pathogenesis and progression of glaucoma. The triglyceride-glucose (TyG) index, a simple and reliable surrogate marker for insulin resistance, has been widely used in metabolic and cardiovascular research. However, its relevance to glaucoma remains largely unexplored. This study aims to investigate the association between the TyG index and glaucoma, thereby providing new insights into the metabolic mechanisms underlying this vision-threatening disease. A cross-sectional analysis of the 2005 to 2008 National Health and Nutrition Examination Survey was performed. The 2005 to 2008 National Health and Nutrition Examination Survey national household survey for adults ≥ 40 years was examined for glaucoma patients. TyG index was calculated as ln [fasting triglyceride (mg/dL) × fasting glucose (mg/dL)/2]. A multifactorial logistic regression model was used to investigate the association between TyG index and glaucoma. Restricted multivariate logistic regression model and cubic spline analysis was used to assess the association between TyG index and risk of glaucoma. A total of 973 patients were included. Higher TyG indices were significantly associated with an increase in glaucoma in U.S. adults. TyG index is positively associated with the risk of developing glaucoma. TyG index may be a therapeutic target and an important predictor of glaucoma.

## 1. Introduction

Glaucoma is a group of eye diseases characterized by progressive optic nerve damage, often associated with increased intraocular pressure (IOP), and is a leading cause of irreversible blindness worldwide.^[1]^ The global prevalence of glaucoma among middle-aged and elderly individuals is approximately 3.5%, with primary open-angle glaucoma having a higher prevalence than primary closed-angle glaucoma.^[2]^ For a long time, high IOP has been considered as an important risk factor for glaucoma.^[3,4]^ Patients with high IOP usually have abnormal aqueous circulation in their eyes, which leads to optic nerve damage and glaucoma.^[5,6]^ The pathological features include retinal ganglion cell loss, thinning of the neuroretinal rim, and changes in the optic disc, which collectively lead to visual field defects and, ultimately, permanent blindness.^[7]^

The pathogenesis and progression of glaucoma are also closely associated with inflammation.^[8–10]^ Initial insults, such as elevated IOP or ischemia, can induce a para-inflammatory response in the optic nerve and retina, representing an adaptive immune reaction that helps maintain tissue homeostasis.^[11,12]^ Within the anterior chamber angle, inflammatory mediators contribute to disease progression by altering the extracellular matrix metabolism of trabecular meshwork cells, thereby increasing aqueous humor outflow resistance and elevating IOP.^[13]^ However, under persistent stressors (including mechanical strain, oxidative stress, and cellular dysfunction) this para-inflammatory balance becomes disrupted and shifts toward a chronic pro-inflammatory state, leading to microglial activation and the release of inflammatory mediators, which in turn exacerbate retinal ganglion cell apoptosis.^[14]^

Prior research has shown that elevated triglyceride-glucose index (TyG) levels are associated with systemic conditions, including those that are also known risk factors for glaucoma.^[15,16]^ The TyG index, a simple and noninvasive indicator of insulin resistance (IR), has been extensively studied in the context of metabolic and cardiovascular diseases.^[17–19]^ Studies have shown that the TyG index and systemic inflammation are both closely associated with cardiovascular disease risk.^[20–22]^ Concurrent elevations of TyG and high-sensitivity C-reactive protein significantly increase the incidence of cardiovascular events and exhibit mutual mediation effects^[23]^; however, their synergistic role remains unclear. Insulin resistance is hypothesized to contribute to glaucomatous damage through mechanisms such as impaired vascular autoregulation, endothelial dysfunction, and increased oxidative stress. These pathophysiological changes may compromise optic nerve perfusion and exacerbate IOP-independent pathways, ultimately increasing the risk of glaucoma.

Genetic predisposition and age remain established risk factors for the onset of glaucoma,^[24]^ but emerging evidence suggests that diabetes may also play a significant role, potentially through mechanisms such as alterations in optic nerve blood flow and increased oxidative stress.^[25]^ In recent years, TyG index, as an index to evaluate IR, has been gradually applied to the study of metabolic diseases.^[26–30]^ The TyG index, calculated using fasting triglyceride and glucose levels, is significantly correlated with IR and serves as an indicator of insulin sensitivity.^[31]^In addition, metabolic syndrome (such as obesity and IR) is also considered to be closely related to the occurrence and development of glaucoma.^[32–35]^ Studies have shown that the TyG index is associated with hypertension, diabetes, and cardiovascular diseases^[17,20,36]^; therefore, understanding the relationship between metabolic biomarkers and glaucoma is crucial for identifying new therapeutic strategies and risk factors. However, its potential relationship with glaucoma remains insufficiently explored. Therefore, we utilized data from 2005 to 2008 National Health and Nutrition Examination Survey (NHANES) to investigate the potential association between the TyG index and glaucoma.

## 2. Materials and methods

### 2.1. Study methods

**Figure 1.**
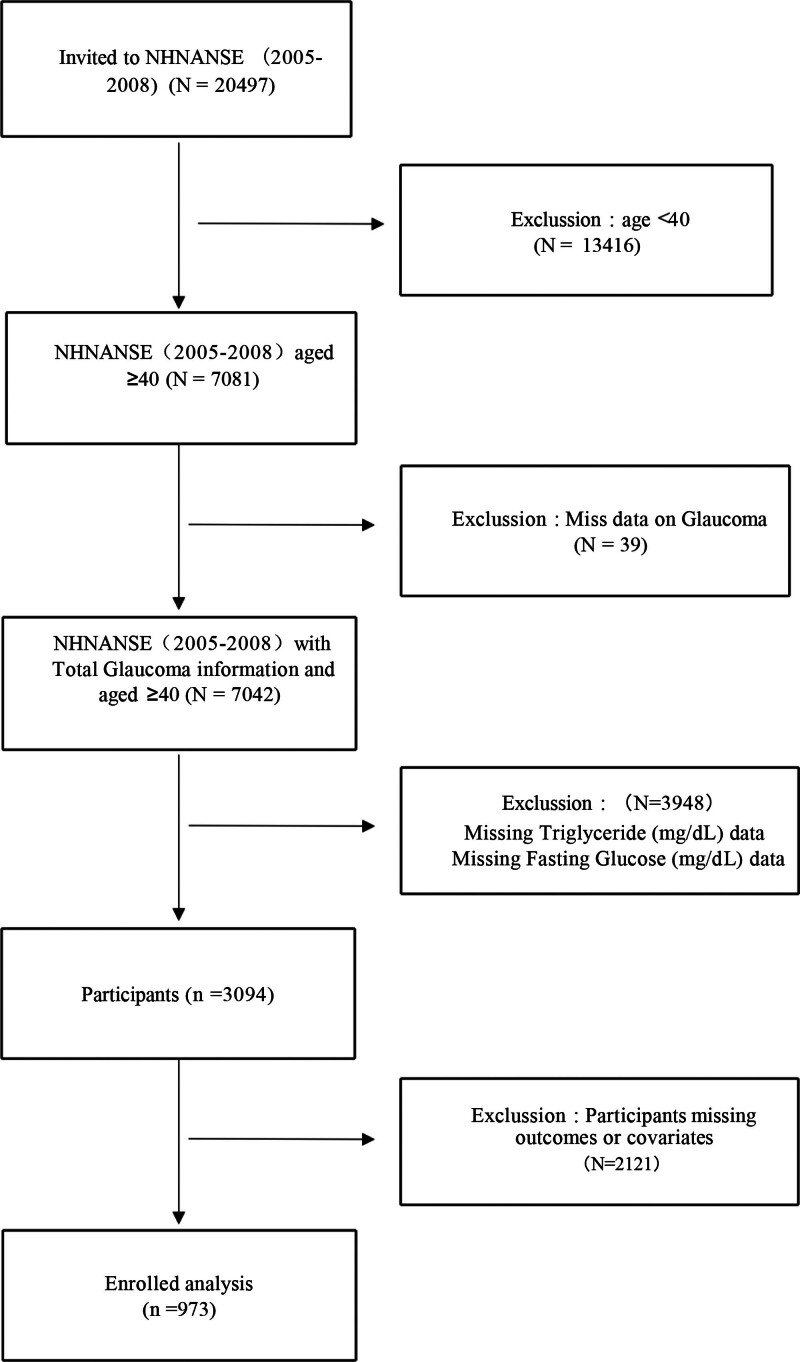
Flow chart of subject selection.

This cross-sectional study analyzed data from the NHANES database covering the period from 2005 to 2008, with a total sample size of 13,350 participants. The high quality and reliability of the data were further ensured by medical, dental, physiological measurements, and laboratory tests conducted by trained professionals. Additionally, the study was approved by an institutional review board, and participants were compensated and provided with medical reports to enhance their willingness to participate and the validity of the data. The detailed study design and procedures are shown in Figure 1.

### 2.2. Defining criteria for glaucoma

The interviewer asked participants if they had glaucoma by asking the following question: “Did the ophthalmologist tell them they had glaucoma?” The responses to this question were “yes,” “no,” “don’t know,” and “missing.” Answers with “don’t know” and “missing” were removed for further research.

### 2.3. Exposure and outcome definitions

The TyG index,^[37]^ used as the exposure variable, is calculated using the formula ln (triglycerides (mg/dL) × fasting glucose (mg/dL))/2. By multiplying these 2 values and taking the natural logarithm, it provides a simpler, more accessible way to gauge metabolic health. In subsequent analyses, the TyG index is treated as a continuous variable and divided into quartiles for more detailed investigation.

### 2.4. Assessment of covariates

Covariates included demographic characteristics, anthropometric measures, and laboratory data, as described in detail below: age, sex (male and female), racial/ethnic categories (non-Hispanic White, non-Hispanic Black, and other races), educational attainment (less than high school and high school or above), and body mass index (BMI), categorized into 3 groups: normal (<18.5 kg/m²), overweight (18.5–24 kg/m²), and obese (≥24 kg/m²). Marital status was classified into 3 categories: never married, married or living with a partner, and widowed, divorced, or separated. Smoking status was determined based on whether participants reported smoking more than 100 cigarettes in their lifetime and their current smoking status. Alcohol drinkers were defined as individuals who consumed at least 12 alcoholic beverages per year. Moderate physical activity was assessed by whether participants engaged in moderate activities in the past 30 days. Other potential confounding variables in this study included age (years), race, sex, education level, BMI status, drinking status, smoking status, coronary heart disease, liver condition, diabetes, HDL cholesterol (mg/dL), moderate recreational activities, dietary health status, triglyceride (mg/dL), and fasting glucose (mg/dL).

### 2.5. Statistical analysis

Continuous variables were described using the mean and standard deviation if normally distributed; otherwise, the median and interquartile range were employed. For normally distributed variables, between-group comparisons were performed using the *t* test; for non-normally distributed variables, the Mann–Whitney *U* test was applied. Categorical variables were expressed as percentages, and the *χ*² test was used for between-group comparisons. Multivariate logistic regression was employed to evaluate the association between the TyG index and self-reported glaucoma. Model 1 adjusted for age, sex, and race. The final model included adjustments for age (years), race, sex, education level, BMI, smoking and drinking status, coronary heart disease, liver conditions, diabetes, HDL cholesterol (mg/dL), moderate recreational activity, dietary health status, triglyceride levels (mg/dL), and fasting glucose (mg/dL). To further validate this relationship, the TyG index was also converted into a categorical variable, and an analysis using the interquartile method was performed. Trend *P*-values were calculated to assess the potential for nonlinearity. *P* < .05 was statistically significant. This study is based on the statistical analysis of DecisionLink.1.0.^[38]^ (DecisionLinnc Core Team.(2023).DecisionLinnc.1.0. https://www.statsape.com).

## 3. Results

### 3.1. Characteristics of the study population

Figure 1 (flow chart of subject selection) is a flow chart of participant screening. There were 7081 individuals ≥ 40 years of age who were screened for glaucoma at NHANES from 2005 through 2008. We excluded those with Miss data on glaucoma (N = 39), missing triglyceride (mg/dL) data, and missing fasting glucose (mg/dL) data (N = 3948), participants missing outcomes or covariates (N = 2121). Finally, a total of 973 eligible participants were included for further analysis.

### 3.2. Description of baseline information of the study sample

Baseline information for all participants in this study is shown in Table 1. This study encompassed 973 participants aged 40 years and older, representing an estimated 17,798,575 U.S. residents. Among these individuals, the mean age was 60.365 ± 13.175 years, and significant differences were observed between participants with and without glaucoma in terms of age, hypertension, and diabetes; however, no significant differences were found in terms of gender, race, education level, marital status, smoking status, and BMI.

**Table 1:** Demographic and clinical characteristics of glaucoma as reported in the 2005 to 2008 United States National Health and Nutrition Examination Surveys.

Characteristic	Participants (N = 973)	No glaucoma (N = 899)	Glaucoma (N = 74)	*P*-value
Age (yr)	60.365 ± 13.175	59.382 ± 12.949	72.311 ± 9.596	<.001
BMI (kg/m^2^)	29.599 ± 7.668	29.626 ± 7.719	29.263 ± 7.068	.696
Cholesterol (mg/dL)	114.621 ± 42.692	114.499 ± 43.552	116.095 ± 30.53	.758
Fasting blood glucose (mg/dL)	57.089 ± 18.008	57.121 ± 18.275	56.703 ± 14.469	.848
Triglyceride (mg/dL)	142.175 ± 126.092	142.21 ± 129.773	141.743 ± 67.318	.976
Gender				.393
Male, n (%)	579 (59.51)	531 (59.07)	48 (64.86)	
Female, n (%)	394 (40.49)	368 (40.93)	26 (35.14)	
Race				.913
Non-Hispanic White, n (%)	512 (52.62)	472 (52.50)	40 (54.05)	
Non-Hispanic Black, n (%)	284 (29.19)	264 (29.37)	20 (27.03)	
Other races, n (%)	177 (18.19)	163 (18.13)	14 (18.92)	
Education level				.343
<9th Grade/9–11th grade, n (%)	227 (23.33)	207 (23.03)	20 (27.03)	
High School Grad/GED or equivalent, n (%)	272 (27.95)	248 (27.59)	24 (32.43)	
Some college or AA degree/college graduate or above, n (%)	474 (48.72)	444 (49.39)	30 (40.54)	
Marital				.089
Never married, n (%)	157 (16.14)	150 (16.69)	7 (9.46)	
Married/living with partner, n (%)	92 (9.46)	88 (9.79)	4 (5.41)	
Widowed/divorced/separated, n (%)	724 (74.41)	661 (73.53)	63 (85.14)	
BMI (kg/m^2^), n (%)				.390
<18.5	15 (1.54)	13 (1.45)	2 (2.70)	
18.5 ≤ BMI < 24	180 (18.50)	163 (18.13)	17 (22.97)	
≥24	778 (79.96)	723 (80.42)	55 (74.32)	
Drinking, n (%)				.146
No	314 (32.27)	284 (31.59)	30 (40.54)	
Yes	659 (67.73)	615 (68.41)	44 (59.46)	
Hypertension, n (%)				<.001
No	495 (50.87)	472 (52.50)	23 (31.08)	
Yes	478 (49.13)	427 (47.50)	51 (68.92)	
Liver condition, n (%)				1.000
No	922 (94.76)	852 (94.77)	70 (94.59)	
Yes	51 (5.24)	47 (5.23)	4 (5.41)	
Chest pain, n (%)				.811
No	689 (70.81)	638 (70.97)	51 (68.92)	
Yes	284 (29.19)	261 (29.03)	23 (31.08)	
Smoking, n (%)				.819
No	415 (42.65)	382 (42.49)	33 (44.59)	
Yes	558 (57.35)	517 (57.51)	41 (55.41)	
Healthy diet, n (%)				.637
Fair	211 (21.69)	198 (22.02)	13 (17.57)	
Poor	71 (7.30)	66 (7.34)	5 (6.76)	
Good/very good/excellent	691 (71.02)	635 (70.63)	56 (75.68)	
Moderate activity, n (%)				.691
No	577 (59.30)	531 (59.07)	46 (62.16)	
Yes	396 (40.70)	368 (40.93)	28 (37.84)	
Diabetes, n (%)				.035
Yes	160 (16.44)	139 (15.46)	21 (28.38)	
No	788 (80.99)	736 (81.87)	52 (70.27)	
Borderline	23 (2.36)	22 (2.45)	1 (1.35)	

BMI = body mass index, NHANES = National Health and Nutrition Examination Survey.

To further investigate the correlation between TyG index and glaucoma, Table 2 presents the baseline characteristics of patients divided into quartiles according to TyG index. TyG index was divided into 4 groups in Q1 (<8.22), Q2 (8.22–8.62), Q3 (8.62–9.06), and Q4 (>9.06). Individuals with higher BMI in Q4, age, ethnicity, male patients, hypertension, smoking history, prevalence of diabetes differed significantly (*P* < .001) between Q1, Q2, Q3, and Q4. The prevalence of hypertension increased across TyG quartiles (37.79%, 43.50%, 51.09%, and 59.14%, respectively; *P* for trend < .001), showing a significant positive association between higher TyG index and hypertension. However, Q2 accounted for the highest proportion (80.08%) among the 4 groups with favorable dietary conditions.

**Table 2:** Relationship between glaucoma and TYG index in different subgroups in NHANES 2005 to 2008.

Characteristics	Quantile 1 < 8.22	Quantile 2 8.22–8.62	Quantile 3 8.62–9.06	Quantile 4 >9.06	*P*
Age (yr)	57.064 ± 13.196	60.805 ± 13.015	61.54 ± 13.317	60.849 ± 12.908	.003
BMI (kg/m^2^)	27.453 ± 6.334	27.926 ± 6.176	30.258 ± 9.134	31.745 ± 7.376	<.001
Cholesterol (mg/dL)	96.25 ± 11.463	101.459 ± 12.135	109.214 ± 23.061	142.9 ± 66.649	<.001
Fasting blood glucose (mg/dL)	69.128 ± 20.762	63.28 ± 17.805	54.576 ± 13.486	46.695 ± 13.081	<.001
Triglyceride (mg/dL)	59.488 ± 13.199	92.22 ± 14.058	130.533 ± 25.826	248.713 ± 191.533	<.001
Gender					.045
Male, n (%)	115 (66.86)	152 (61.79)	160 (57.97)	152 (54.48)	
Female, n (%)	57 (33.14)	94 (38.21)	116 (42.03)	127 (45.52)	
Race					<.001
Non-Hispanic White, n (%)	78 (45.35)	125 (50.81)	158 (57.25)	151 (54.12)	
Non-Hispanic Black, n (%)	82 (47.67)	80 (32.52)	62 (22.46)	60 (21.51)	
Other races, n (%)	12 (6.98)	41 (16.67)	56 (20.29)	68 (24.37)	
Education level					.116
<9th grade/9–11th grade, n (%)	47 (27.33)	43 (17.48)	66 (23.91)	71 (25.45)	
High School Grad/GED or equivalent, n (%)	40 (23.26)	68 (27.64)	82 (29.71)	82 (29.39)	
Some college or AA degree/college graduate or above, n (%)	85 (49.42)	135 (54.88)	128 (46.38)	126 (45.16)	
Marital					.301
Never married, n (%)	36 (20.93)	37 (15.04)	42 (15.22)	42 (15.05)	
Married/living with partner, n (%)	21 (12.21)	22 (8.94)	21 (7.61)	28 (10.04)	
Widowed/divorced/separated, n (%)	115 (66.86)	187 (76.02)	213 (77.17)	209 (74.91)	
Drinking, n (%)					.372
No	65 (37.79)	77 (31.30)	83 (30.07)	89 (31.90)	
Yes	107 (62.21)	169 (68.70)	193 (69.93)	190 (68.10)	
Hypertension, n (%)					<.001
No	107 (62.21)	139 (56.50)	135 (48.91)	114 (40.86)	
Yes	65 (37.79)	107 (43.50)	141 (51.09)	165 (59.14)	
Liver condition, n (%)					.527
No	163 (94.77)	231 (93.90)	266 (96.38)	262 (93.91)	
Yes	9 (5.23)	15 (6.10)	10 (3.62)	17 (6.09)	
Chest pain, n (%)					.192
No	120 (69.77)	181 (73.58)	203 (73.55)	185 (66.31)	
Yes	52 (30.23)	65 (26.42)	73 (26.45)	94 (33.69)	
Smoking, n (%)					.027
No	90 (52.33)	104 (42.28)	115 (41.67)	106 (37.99)	
Yes	82 (47.67)	142 (57.72)	161 (58.33)	173 (62.01)	
Diabetes, n (%)					<.001
Yes	14 (8.14)	18 (7.32)	33 (11.96)	95 (34.05)	
No	155 (90.12)	226 (91.87)	235 (85.14)	172 (61.65)	
Borderline	3 (1.74)	1 (0.41)	7 (2.54)	12 (4.30)	
Healthy diet, n (%)					.021
Fair	42 (24.42)	35 (14.23)	67 (24.28)	67 (24.01)	
Poor	12 (6.98)	14 (5.69)	19 (6.88)	26 (9.32)	
Good/very good/excellent	118 (68.60)	197 (80.08)	190 (68.84)	186 (66.67)	
Moderate activity, n (%)					.803
No	104 (60.47)	140 (56.91)	163 (59.06)	170 (60.93)	
Yes	68 (39.53)	106 (43.09)	113 (40.94)	109 (39.07)	

BMI = body mass index, NHANES = National Health and Nutrition Examination Survey, TyG = triglyceride-glucose index.

### 3.3. Association between TyG and the presence of glaucoma

In this cross-sectional analysis of a nationally representative U.S. adult population, we did not directly establish an association between the TyG index and glaucoma prevalence due to the influence of multiple confounding factors (e.g., race and age) and the inability to determine the temporal sequence between them. Consequently, causal inference could not be made, and statistical significance was achieved only after full adjustment for these confounders. The association between TYG levels and glaucoma is shown in Table 3. Elevated levels of the TyG index are associated with an increased risk of glaucoma.

**Table 3:** Weighted relationship between triglyceride-glucose index and glaucoma.

TyG	Model 1	*P*-value	Model 2	*P*-value	Model 3	*P*-value
Glaucoma						
TyG index	1.22 (0.86, 1.71)	.237	1.27 (0.85, 1.88)	.223	3.79 (0.98, 20.53)	.086
TyG index quartile						
Quantile 1 < 8.22	1.00 (Ref)		1.00 (Ref)		1.00 (Ref)	
Quantile 2 8.22–8.62	0.92 (0.43, 1.97)	.848	0.80 (0.36, 1.76)	.586	1.01 (0.43, 2.37)	.977
Quantile 3 8.62–9.06	1.28 (0.64, 2.63)	.477	1.08 (0.51, 2.31)	.826	1.57 (0.63, 4.12)	.340
Quantile 4 >9.06	1.81 (0.94, 3.59)	.078	1.81 (0.91, 3.79)	.094	3.98 (1.20, 15.08)	.031
*P* for trend	.042		.049		.041	

In the sensitivity analysis, TyG was converted from a continuous variable to a categorical variable (quartiles).

Data are presented as odds ratios, 95% confidence intervals, *P*-value and *P* for trend.

Model 1: non-adjusted.

Model 2 adjusted for: age (years); race; sex.

Model 3: adjusted for age (years); race; sex; education level; BMI status; drinking status; smoking status; coronary heart disease; liver condition; diabetes; HDL cholesterol (mg/dL); moderate recreational activities; dietary health status; triglyceride (mg/dL); fasting glucose (mg/dL).

TyG = triglyceride-glucose index.

#### 3.3.1. Analysis of the TyG index as a continuous variable

In the weighted logistic regression analyses, no significant association was observed between the continuous TyG index and glaucoma in the unadjusted and partially adjusted models (Model 1: OR = 1.22, 95% CI: 0.86–1.71, *P* = .237; Model 2: OR = 1.27, 95% CI: 0.85–1.88, *P* = .223). In the unadjusted model (Model 1), the association between TyG index and glaucoma risk did not reach statistical significance (OR = 1.22, 95% CI: 0.86–1.71, *P* = .237). In Model 2, after partial adjustment for the covariates of age, race, and sex, the OR increased slightly to 1.27 (95% CI: 0.85–1.88, *P* = .223), but still did not reach a significant level. However, in Model 3, after full adjustment for covariates including education level, BMI, lifestyle factors, and metabolism-related indicators, the association strengthened considerably, with the TyG index showing a borderline significant relationship with glaucoma risk (Model 3: OR = 3.79, 95% CI: 0.98–20.53, *P* = .086). The trend tests across TyG quartiles were statistically significant in all models (*P* for trend = .042, .049, and .041, respectively), supporting a consistent monotonic dose–response relationship between higher TyG index and increased glaucoma prevalence. Although the comparison of Q4 with Q1 did not reach statistical significance in Models 1 and 2, the trend analysis (by incorporating risk changes across all quartiles) more sensitively captured the overall linear association, suggesting a progressive elevation in glaucoma risk with increasing TyG index. These results suggest that the direct association between TyG index and glaucoma was weak when the confounding factors were not adjusted, but the strength of the association was significantly enhanced after comprehensively controlling for metabolism- and lifestyle-related factors, suggesting that TyG index may have an important role in the pathogenesis of glaucoma.

#### 3.3.2. Analysis of the TyG index by quartile grouping

After dividing the TyG index into quartile groups (based on Quantile 1: <8.22), the analysis showed an increasing trend in glaucoma risk with increasing TyG index. In Quantile 4 (>9.06), the OR for both Model 1 and Model 2 was 1.81 (*P* .078 and .094, respectively), which was close to the significant level. In contrast, in the fully adjusted Model 3, the OR for Quantile 4 increased significantly to 3.98 (95% CI: 1.20–15.08, *P* = .031), indicating that individuals with the highest TyG index were at approximately 4 times the risk of developing glaucoma than the baseline group. In addition, trend analysis (*P* for trend) showed a statistically significant trend in the association between TyG quartiles and glaucoma risk in all 3 models (*P* = .042, .049, and .041, respectively). These findings further emphasize the potential causal association between elevated TyG index and glaucoma risk, providing supportive evidence for its use as a predictor of metabolism-related risk.

#### 3.3.3. Restricted triple spline analysis to study the relationship between TyG levels and glaucoma

**Figure 2.**
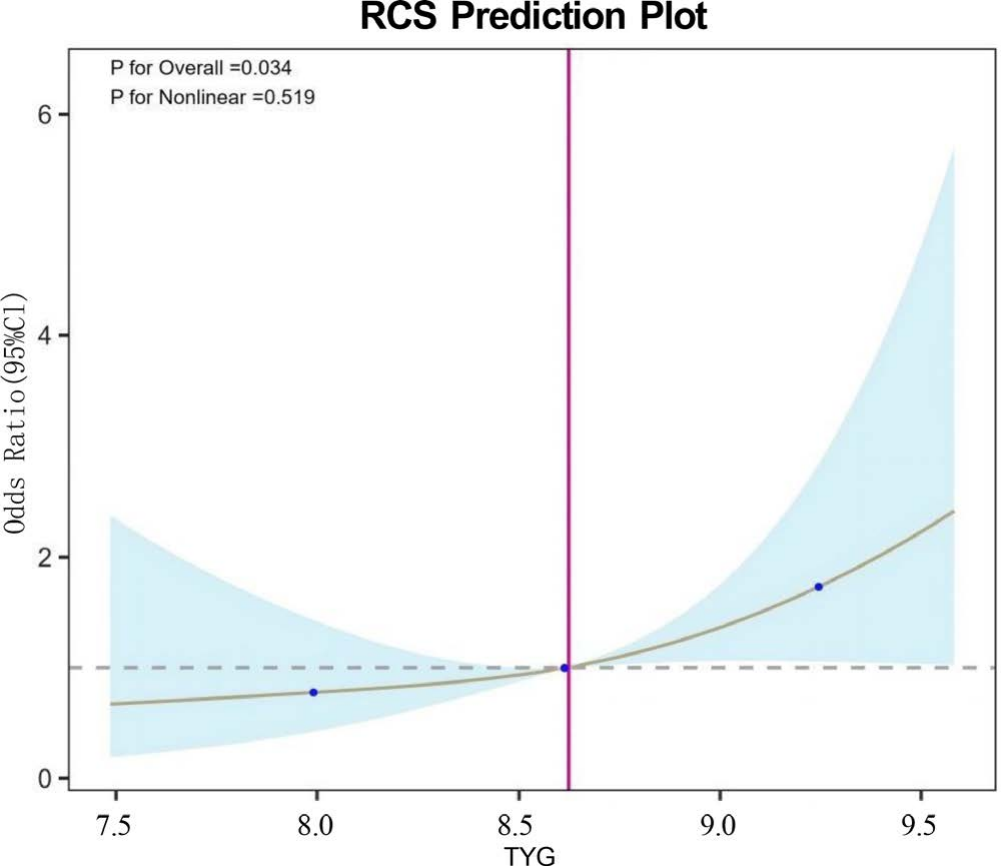
RCS prediction plot.

Based on the fully adjusted Model 3, the RCS analysis presented in Figure 2 demonstrated a statistically significant overall positive association between TyG and the risk of the outcome (*P* for overall = .034), whereas no significant evidence of nonlinearity was observed (*P* for nonlinear = .519). Using TyG = 8.5 as the reference point, the risk was slightly lower below this threshold, gradually increased above it, and became more pronounced when TyG exceeded 9.0. Overall, the relationship between TyG and outcome risk was more consistent with a linear increasing trend rather than a nonlinear pattern.

### 3.4. Subgroup analyses

**Figure 3.**
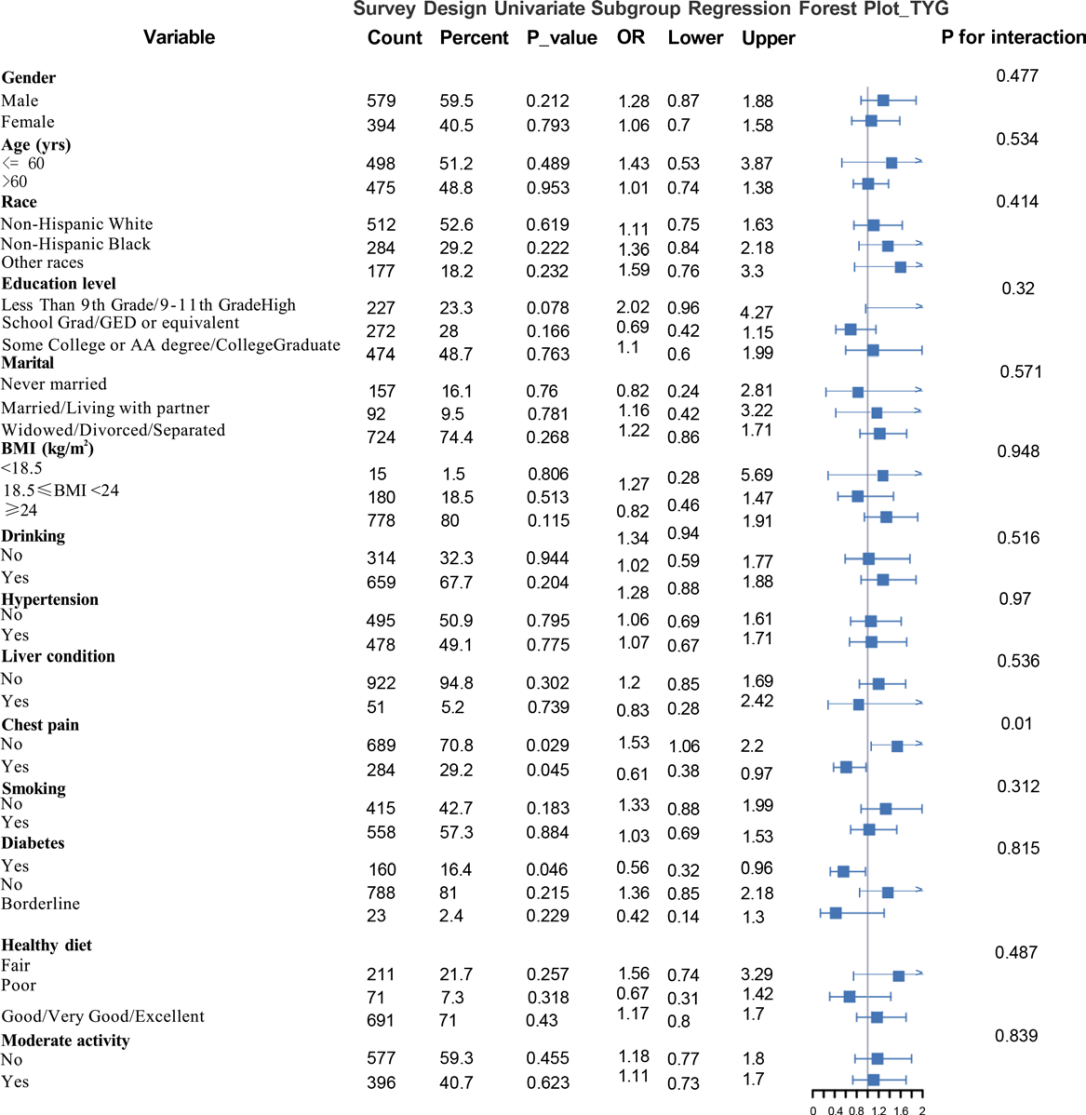
Survey design univariate subgroup regression forest plot – TyG.

**Figure 4.**
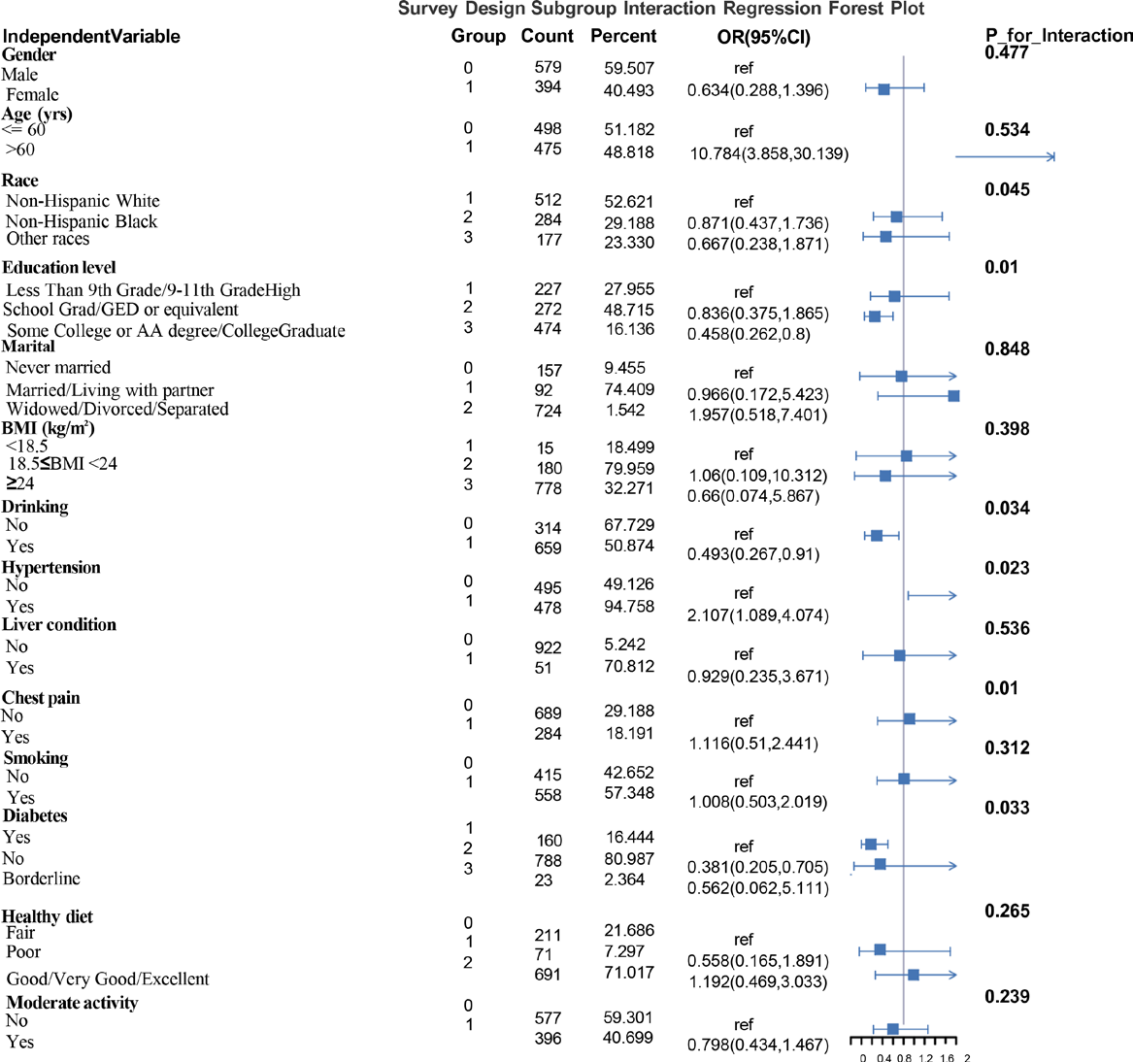
Subgroup interaction regression forest plot.

We performed 1-way subgroup regression analyses and multifactor subgroup interaction regression analyses to assess the potential moderating effects of various stratification variables. One-way subgroup regression analysis in Figure 3 (Survey Design Univariate Subgroup Regression Forest Plot – TyG) without adjusting for confounders found that those without diabetes had a significantly lower risk (OR = 0.56, 95% CI: 0.32–0.96, *P* = .046) suggesting that diabetes may be a risk factor, and that chest pain (*P* = .029) showed a significant association suggesting that chest pain was a significant predictor of the outcome; the status of chest pain (*P* for Interaction = .01) indicated a significant interaction with other variables, that is, chest pain affects TyG differently in different populations and may be a key moderator variable. Smokers (OR = 1.33), although the *P*-value was not significant, the OR suggests that there may be a trend for smokers. Subgroup interaction regression analysis after adjusting for confounders found. The association between TyG and glaucoma across subgroups in Figure 4 (Survey Design Subgroup Interaction Regression Forest Plot) was found to be as high as an OR of 10.784 (95% CI: 3.858–30.139) for the age > 60 years group, suggesting that the risk was significantly elevated in this group (*P* = .534 was not significant, but the OR suggests a strong association). Non-Hispanic Blacks (OR = 0.871) and all other races (OR = 0.667) had a lower risk than non-Hispanic Whites, but the interaction *P*-value of .045 (<.05) suggests a significant interaction effect between races. The risk was significantly lower in the college and higher education (OR = 0.458, 95% CI: 0.262–0.8) than in the lower education group (*P* for interaction = .01). Those without diabetes had a significantly lower risk (OR = 0.381, 95% CI: 0.205–0.705, *P* = .033). Those with hypertension had a significantly higher risk (OR = 2.107, 95% CI: 1.089–4.074, *P* = .023 is significant, but OR suggests potential association). Alcohol drinkers had a reduced risk (OR = 0.493, 95% CI: 0.267–0.91, *P* = .516 not significant).

## 4. Discussion

This study explored the association between the TyG index and glaucoma using data from the NHANES database. A key limitation of this study lies in the potential imprecision of glaucoma diagnosis within the NHANES dataset, which may rely on self-reported information or basic screening procedures rather than comprehensive ophthalmologic evaluations. Non-differential misclassification could attenuate the observed association between the TyG index and glaucoma, whereas differential misclassification may either overestimate or obscure the true effect. Despite this concern, NHANES provides a robust platform for large-scale, population-based epidemiological research, with its nationally representative sampling framework and extensive covariate data enhancing both statistical power and external validity.

Interestingly, the association between TyG and glaucoma appears to differ from its role in other systemic diseases. Unlike cardiovascular conditions, where TyG is associated with a wide range of outcomes, our findings suggest that TyG’s impact on glaucoma may involve more specific mechanisms.^[20,39]^ For example, the vascular dysregulation and microvascular changes linked to IR could play a pivotal role in glaucoma pathogenesis. These changes may impair the autoregulation of ocular blood flow, reducing perfusion to the optic nerve and exacerbating glaucomatous damage.^[40–42]^ Additionally, systemic inflammation and oxidative stress, both hallmarks of IR, are known contributors to optic nerve vulnerability and retinal ganglion cell apoptosis.^[8,43,44]^

The findings demonstrated that an elevated TyG index is independently associated with a higher risk of glaucoma, with the strongest association observed in individuals in the highest TyG quartile (Q4: TyG > 9.06). This relationship persisted after adjusting for a wide range of confounders, suggesting that the TyG index may serve as a useful marker for identifying individuals at higher risk of developing glaucoma. Our study aligns with these findings, emphasizing a significant trend between rising TyG levels and glaucoma risk. Among the 4 TyG quartiles, the highest quartile (Q4) exhibited a nearly fourfold increase in glaucoma risk compared to the lowest quartile (Q1). This association remained statistically significant after controlling for potential confounders such as age, BMI, smoking, and comorbid conditions. The trend analysis further confirmed the robustness of this association, with the relationship persisting across different statistical models.

Our results also highlight the complexity of TyG’s role in glaucoma risk. While the highest TyG quartile demonstrated a significant association, the effect was not observed in the intermediate quartiles (Q2 and Q3). This may suggest a threshold effect, where the risk of glaucoma becomes markedly elevated only beyond a specific TyG level. Alternatively, the nonlinear association could reflect underlying heterogeneity in the population, including genetic predisposition, comorbid conditions, or unmeasured lifestyle factors. Future studies are needed to clarify whether this threshold represents a biological tipping point or is influenced by external variables.

The findings of this study contribute to a growing body of evidence supporting the role of metabolic health in glaucoma risk. While traditional risk factors for glaucoma, such as age, IOP, and family history, remain crucial, the addition of metabolic markers like TyG could enhance early identification of high-risk individuals. This is particularly important for patients with subclinical metabolic disturbances, who may not yet exhibit overt systemic or ocular symptoms but are already at elevated risk for glaucoma progression.

## 5. Conclusions

In this cross-sectional analysis of a nationally representative U.S. adult population, we observed a positive association between higher TyG index and the presence of glaucoma, which became statistically significant only after full adjustment for multiple confounding variables. However, given the limitations of the cross-sectional design, the potential for residual confounding, and variability in significance across models, these findings should be interpreted with caution. Further prospective cohort studies with more diverse populations and standardized measurement of metabolic indicators are warranted to validate and better understand the potential link between TyG index and glaucoma.

## Author contributions

**Conceptualization:** Zongxiu Luo, Jianye Chen.

**Data curation:** Zongxiu Luo, Lijuan Wei, Jianye Chen.

**Funding acquisition:** Lijuan Wei.

**Formal analysis:** Zongxiu Luo, Lijuan Wei, Jianye Chen.

**Investigation:** Zongxiu Luo, Lijuan Wei, Jianye Chen.

**Methodology:** Zongxiu Luo, Lijuan Wei.

**Project administration:** Lijuan Wei, Jianye Chen.

**Resources:** Jianye Chen.

**Software:** Zongxiu Luo, Jianye Chen.

**Supervision:** Lijuan Wei.

**Validation:** Lijuan Wei.

**Visualization:** Lijuan Wei, Jianye Chen.

**Writing – original draft:** Zongxiu Luo, Jianye Chen.

**Writing – review & editing:** Zongxiu Luo, Jianye Chen.
